# Anxiety and Depression Symptoms of Family Members of Intensive Care Unit Patients: A Prospective Observational Study and the Lived Experiences of the Family Members

**DOI:** 10.1055/s-0043-1769933

**Published:** 2023-06-16

**Authors:** Hande Gurbuz, Nalan Demir

**Affiliations:** 1 Department of Anesthesiology and Reanimation, University of Health Sciences, Bursa School of Medicine, Bursa City Hospital, Bursa, Turkey; 2 Department of Anesthesiology and Reanimation. Formerly: University of Health Sciences, Derince Training and Research Hospital, Kocaeli, Turkey; 3 Department of Chest Diseases, Intensive Care Division, Ankara City Hospital, Ankara, Turkey

**Keywords:** anxiety, intensive care unit, critically ill, death, depression, depressive symptoms, family

## Abstract

**Background** The aim of this study is to investigate the factors affecting symptoms of anxiety and depression in the family members of critically ill patients.

**Methods** This prospective cohort study was conducted in an adult tertiary care mixed medical–surgical intensive care unit (ICU) at a tertiary-level teaching hospital. The symptoms of anxiety and depression of first-degree adult relatives were evaluated with the Hospital Anxiety and Depression Scale. Four family members were interviewed and asked about their experiences during the ICU process.

**Results** A total of 84 patients and their family members were included in the study. The symptoms of anxiety were present in 44/84 (52.4%), and depression was present in 57/84 (67.9%) family members. A nasogastric tube was found to be related to anxiety ( *p* = 0.005) and depressive symptoms ( *p* = 0.002). The family members of the patients with an acute developed illness had 3.9 (95% confidence interval [CI]: 1.4–10.9) times the odds of having the symptoms of anxiety and 6.2 (95% CI: 1.7–21.7) times the odds of having the symptoms of depression than the family members of the patients with an illness developed on a chronic basis. The family members of the patients who died in the ICU had 5.0 (95% CI: 1.0–24.5) times the odds of being depressed than the patients discharged from the ICU. All interviewees stated having difficulty understanding and remembering what was told. The common feelings of all the interviewees were desperation and fear.

**Conclusions** Awareness of the emotional stress of family members can help develop interventions and attitudes to alleviate symptom burden.

## Introduction

While treating and dealing with critically ill patients, families are often neglected. However, the intensive care period is overwhelmingly stressful and exhausting not only for the patients but also for their relatives. The fact that a loved one is critically ill, the thought that the patient might be suffering due to various invasive procedures in the intensive care unit (ICU), the uncertainty of the outcomes of illness, and the fear of losing a loved one will undoubtedly increase the emotional burden on the patient's relatives. 1 Additionally, limited visits and information times, not being involved in the follow-up and treatment process in ICU as it was when at home or in the ward, and being in a decision-making position in some cases, further aggravate this emotional stress on family members. 2 3 Having a critically ill relative in the ICU and thus grinding emotional stress poses a risk for exhibiting posttraumatic stress disorder, anxiety disorder, and depression symptoms according to the level of coping with the stress of the family member. 3 4 It has been reported that when assistance in grief management is not provided to family members, the grief period may extend up to 1 year after the loss. 5 Moreover, even up to 40% of the relatives of the patients may show symptoms of generalized anxiety, major depressive disorder, or complicated grief 1 year after the loss. 5

After recognizing the psychological repercussions of the closest family members of dying ICU patients, patient-centered care rather than clinician- or disease-centered care, based on the view that not only the patients being treated but also their relatives have some needs, has gained prominence. 6 7 8 9 10 Previous studies indicated that at least two-thirds of the family members of ICU patients experience anxiety or depression symptoms during the ICU period. 2 It has been reported that these symptoms can be affected by age, gender, education level, relationship, and disease severity. 2 11 Anxiety, depression, and stress disorder symptoms, but not the syndrome, can be measured quantitatively with some validated tools.

In this study, we aimed to investigate the factors affecting symptoms of anxiety and depression in the family members of the patients in a tertiary care ICU before the coronavirus outbreak. Interviews with the family members were included to understand better their lived experiences.

## Methods

This prospective observational cohort study was held in an adult tertiary mixed medical–surgical ICU at a tertiary-level teaching hospital. The study was conducted in accordance with the Helsinki Declaration and reported in adherence to the Strengthening the Reporting of Observational Studies in Epidemiology guidelines for cross-sectional studies.

## Patient Selection

### Inclusion and Exclusion Criteria

All the adult patients admitted to the ICU for any reason between October 2019 and March 2020 (before the outbreak arrangements) and who were anticipated to stay in ICU for more than 2 days were evaluated for inclusion in the study. The patients discharged from the ICU for any reason before 48 hours were excluded from the study.

### Family Survey

Following the daily face-to-face information about the patient's treatment process by the intensivist, the first-degree adult relatives were allowed to pay a bedside visit to the patient in the ICU once a day for about 15 minutes. After their visits on the second day of admission, the first-degree adult relatives were asked to complete the survey. The survey proposed to the next-of-kin included the validated Turkish version of the Hospital Anxiety and Depression Scale (HADS). 12

### Hospital Anxiety and Depression Scale

The HADS was first developed to assess emotional disorders in hospitalized patients. 13 The scale is a basic, easily applicable, valid, and reliable screening tool to detect the symptoms of mood disorders used not only in patients but also in the relatives of the patients. 1 2 3 14 The scale is a 14-item tool, each question ranging from “0” to “3” points regarding the feelings in the last few days. It consists of two parts, each with seven questions, with total scores ranging from 0 to 21 to determine the severity of anxiety and depression symptoms. The HADS has been validated in Turkish, with a cutoff score of 7 for the depression subscale and 10 for the anxiety subscale. 12 Thus, HADS scores of more than or equal to 8 for the depression subscale and more than or equal to 11 for the anxiety subscale were indicated as positive for the related disorder in this study, and then the results of the HADS were dichotomized accordingly.

## Data Collection

### ICU Patients

Characteristics of the patients, including the data regarding the illness, were recorded. The Acute Physiology and Chronic Health Evaluation (APACHE-II) scores and estimated mortality rates were calculated after 24 hours of ICU admission. 15 The tubes and catheters that the patients had during the family member's visit were recorded to detect how these interventions affected the family member's mood state. Finally, the outcome of the patients (discharged to the ward or another clinic and death) was noted.

### Family Members

The family members' sociodemographic characteristics, including age, gender, relationship to the patient, and education level, were collected.

## Interviews with the Family Members

This study does not follow a structured phenomenological method. 16 Purposeful sampling was employed to evaluate how specific characteristics of the patients and the family members affect their mood state. During the study, before data analysis, the assumption was that the chronicity of the illness, and the family members' familiarity with the ICU environment would affect the mood state of the relatives. Therefore, family members of four patients, as the representatives of each assumption, were selected to be interviewed. These four patients had high calculated mortality rates (>70%) and APACHE-II scores (≥30). 15 The participants who accepted the interview were the family members who also took the survey and were found to have both anxiety and depressive symptoms.

The interviews were done at any time during the hospitalization of the patients. The open-ended interviews lasted about 60 minutes each and were conducted in the family members' homes or a quiet room near the ICU. The participants were asked to talk about their experiences and feelings as the closest family member of the patient. No cameras, voice tape, or video recorders were used.

### Ethical Approval

The protocol was approved by the institutional ethics committee (Derince Training and Research Hospital, date: 12.02.2019, decree: 2019/131). Informed consent for the survey and interviews were obtained.

### Statistics

The statistical data were analyzed using SPSS Statistics for Windows version 20.0, 2011 (IBM Corp., Armonk, New York, United States). The normality of distribution was determined with the Shapiro–Wilk test. Non-normally distributed continuous variables were tested using the Mann–Whitney-U test. The Pearson chi-squared or Fisher's exact (where appropriate) tests were used to analyzing the categorical data.

The independent factors of anxiety and depression were evaluated by building a logistic regression model. The statistically significant variables affecting the presence of anxiety and depression were included in the multivariate analysis. Before the regression analysis, redundant variables were eliminated by running a multicollinearity test, and the model's goodness of fit was analyzed using the Hosmer–Lemeshow test.

The results were presented as mean ± standard deviation (SD), median (25-75 percentiles), and numbers (percent). The effect sizes were shown as odds ratios and 95% confidence intervals (CIs). Bonferroni correction was used for multiple comparisons (education level and relationship). An alpha value of less than 0.05 was considered statistically significant.

The sample size of the study was calculated using the G*Power (version 3.1.9.4) based on a previous study, in which the mean ± SD anxiety score of the first-degree relatives of the ICU patients was found to be 9.49 ± 4.18. 11 Accordingly, a minimum number of 84 patients were needed to detect a significant level of anxiety more than or equal to 11 points, assuming an alpha error of 0.05, power of 0.9, an effect size of 0.36, and a two-sided alternative hypothesis. Participants were included in the study using the quota sampling method consecutively from the initiation date of the study until 84 patient quotas were filled according to the inclusion and exclusion criteria.

## Results

The diagnoses and interventions of the patients are presented in Tables 1 and 2 . The demographics of the patients and next-of-kin according to the presence of anxiety and depression symptoms are presented in Table 3 . The mean anxiety score of all participating family members was 10.5 ± 4.2, and the depression score was 9.7 ± 4.6 points (not presented in the table). Forty-four (52.4%) next-of-kin had anxiety, and 57 (67.9%) had depression symptoms. The relatives of the patients who had a nasogastric tube showed statistically significantly high anxiety ( *p* = 0.005) and depression symptoms ( *p* = 0.002) ( Table 2 ). There was no statistically significant difference in anxiety and depression symptoms for the remaining interventions.

**Table 1:** Diagnoses of the patients on admission

Diagnoses	*n* (%)
Cerebrovascular disease	20 (23.8)
Cancer surgery	12 (14.3)
Respiratory failure (pneumonia, COPD)	11 (13.1)
Post CPR	10 (11.9)
Trauma	8 (9.5)
Major abdominal surgery (bowel obstruction, gastrointestinal bleeding)	6 (7.1)
Cancer	5 (6.0)
Myocardial infarction	5 (6.0)
Renal failure	3 (3.6)
Miscellaneous (sepsis, cirrhosis, etc.)	4 (4.8)

Abbreviations: COPD, chronic obstructive pulmonary disease; CPR, cardiopulmonary resuscitation.

**Table 2:** ICU interventions

Interventions	*n* (%)	Anxiety		*p* -Value	Depression		*p* -Value
		+ ( *n* = 44)	− ( *n* = 40)		+ ( *n* = 57)	− ( *n* = 27)	
Urinary catheter	84 (100)	44 (100)	40 (100)	N/A	57 (100)	27 (100)	N/A
Nasogastric tube	64 (76.2)	39 (60.9)	25 (39.1)	0.005 b	49 (76.6)	15 (23.4)	0.002 b
Central venous catheter	64 (76.2)	37 (57.8)	27 (42.2)	0.075	45 (70.3)	19 (29.7)	0.389
Endotracheal tube/tracheostomy tube	50 (59.5)	30 (60.0)	20 (40.0)	0.090	37 (74.0)	13 (26.0)	0.144
Surgical drain	41 (48.8)	23 (43.9)	18 (56.1)	0.505	27 (65.9)	14 (34.1)	0.701
Noninvasive mechanic ventilation	15 (17.9)	10 (66.7)	5 (33.3)	0.222	11 (73.3)	4 (26.7)	0.616
Hemodialysis catheter	11 (13.1)	5 (54.5)	6 (45.5)	0.622	7 (63.6)	4 (36.4)	0.748
Chest tube a	7 (8.3)	5 (71.4)	2 (28.6)	0.437	5 (71.4)	2 (28.6)	1.000

Abbreviations: ICU, intensive care unit; N/A, not applicable.

Results are presented as *n* (%).

a Fisher's exact test.

b Statistically significant; *p* < 0.05.

**Table 3:** Demographics of the patients and next-of-kin according to anxiety and depression

		Anxiety		*p*	Depression		*p* -Value
		+ ( *n* = 44)	− ( *n* = 40)		+ ( *n* = 57)	− ( *n* = 27)	
Characteristics of the patients	Scale scores	13.5 (12.0––14.8)	8.0 (5.3-9)	N/A	10 (6.8–13.0)	6.0 (4.0–6.0)	N/A
	Female/Male	19/25 (46.3/58.1)	22/18 (53.7/41.9)	0.279	27/30 (65.9/69.8)	14/13 (34.1/30.2)	0.701
	Age	65.0 (50.5-76.8)	67.0 (54.3-77.5)	0.680	65.0 (53.5-78.0)	67.0 (54.0-76.0)	0.642
	APACHE-II	24.0 (13.0–31.3)	15.5 (10.0–22.5)	0.004 a	23.0 (13.0–29.0)	14.0 (8.0–20.0)	0.001 a
	Calculated mortality rate	32.5 (15.5–55.8)	20.0 (12.0–30.8)	0.013 a	30.0 (16.0–55.0)	18.0 (8.0–28.0)	0.004 a
	ICU stay time	10.0 (6.0–32.5)	8.0 (4.0–22.5)	0.097	10.0 (5.5–28.5)	6.0 (3.0–23.0)	0.151
	Perioperative / medical treatment	18/26 (50.0/54.2)	18/22 (50.0/45.8)	0.705	22/35 (61.1/72.9)	14/13 (38.9/27.1)	0.252
	Previous ICU admission, yes/no	22/22 (55.0/50.0)	18/22 (45.0/50.0)	0.647	28/29 (70.0/65.9)	12/15 (30.0/34.1)	0.688
	Acute/chronic disease	34/10 (64.2/32.3)	19/21 (35.8/67.7)	0.005 a	42/15 (79.2/48.4)	11/16 (20.8/51.6)	0.003 a
	Unplanned/planned admission	23/21 (52.3/52.5)	21/19 (47.7/47.5)	0.983	32/25 (72.7/62.5)	12/15 (27.3/37.5)	0.316
	From ED/ward	19/25 (50.0/54.3)	19/21 (50.0/45.7)	0.691	26/31 (68.4/67.4)	12/15 (31.6/32.6)	0.920
	Death/discharged	28/16 (71.8/35.6)	11/29 (28.2/64.4)	0.001 a	35/22 (89.7/48.9)	4/23 (10.3/51.1)	<0.001 a
Characteristics of the next-of-kin	Female/Male	23/21 (51.1/53.8)	22/18 (48.9/46.2)	0.802	30/27 (66.7/69.2)	15/12 (33.3/30.8)	0.802
	Age	47.0 (40.0-52.0)	51.5 (42.0-58.8)	0.074	50.0 (41.5-55.5)	49.0 (41.0-56.0)	0.455
	Previous ICU visit, yes/no	19/25 (50.0/54.3)	19/21 (50.0/45.7)	0.691	27/30 (71.1/65.2)	11/16 (28.9/34.8)	0.569
	Education Primary school Secondary school High school University and higher	12 (40.0) 4 (33.3) 19 (70.4) 9 (60.0)	18 (60.0) 8 (66.7) 8 (29.6) 6 (40.0)	0.059	22 (73.3) 6 (50.0) 20 (74.1) 9 (60.0)	8 (26.7) 6 (50.0) 7 (25.9) 6 (40.0)	0.396 b
	Relationship Children Spouse Parents Siblings	29 (52.7) 11 (57.9) 2 (33.3) 2 (50.0)	26 (47.3) 8 (42.1) 4 (66.7) 2 (50.0)	0.819 b	37 (67.3) 14 (73.7) 4 (66.7) 2 (50.0)	18 (32.7) 5 (26.3) 2 (33.3) 2 (50.0)	0.846 b

Abbreviations: APACHE, Acute Physiology and Chronic Health Evaluation; ED, emergency department; ICU, intensive care unit; N/A, not applicable.

Results are presented as *n* (%) and median (25–75 percentiles).

a Statistically significant, *p* < 0.05.

b Fisher's exact test.

The patients' APACHE-II scores and estimated mortality rates were significantly higher in the family members with anxiety and depression ( *p* < 0.05) ( Table 3 ). Fifty-three (63.1%) patients' illnesses developed as an acute event (unpredictable, without any previously known reasons for the present disease), and 31 (36.9%) patients' illnesses developed over a chronic state of a previously known illness (a predictable course). The family members of the patients who had an acute developed illness had significantly higher rates of anxiety and depression ( *p* < 0.05) ( Table 3 ). The remaining parameters regarding patient and family member characteristics showed no statistical significance.

Forty-five (53.6%) patients were discharged from the ICU, but 39 (46.4%) died in the ICU. Additionally, the presence of anxiety and depression was significantly high among the family members of patients who died in the ICU ( Table 3 ). Even though the patients had yet to die at the time of the survey, this result can be interpreted as the awareness of the approaching death might have led to anxiety and depression. There was no statistically significant difference in anxiety and depression for the family members' educational status and relationship.

Parameters that showed a statistical significance ( *p* < 0.05) were included in the multivariable logistic regression analysis model to analyze the independent factors of anxiety and depression in family members. Among these correlated variables, APACHE-II scores, estimated mortality rates (according to APACHE-II calculation), outcomes (death or discharge), and the state of the illness (acute or chronic) were included in a multicollinearity analysis; accordingly, the estimated mortality rate was excluded due to collinearity with APACHE-II scores (variance inflation factors >3). Based on the Hosmer–Lemeshow test, the models were found to fit well ( *p* = 0.945 for anxiety; *p* = 0.693 for depression). Thus, the family members of the patients with an acute developed illness had 3.9 (95% CI: 1.4–10.9) times the odds of having the symptoms of anxiety and 6.2 (95% CI: 1.7–21.7) times the odds of having the symptoms of depression than the family members of the patients who had an illness developed on a chronic basis. Additionally, the family members of the patients who died in the ICU had 5.0 (95% CI: 1.0–24.5) times the odds of being depressed than the patients discharged from the ICU. ( Table 4 )

**Table 4:** Logistic regression analysis of the factors affecting anxiety and depression

	Wald	Odds ratio	Confidence interval (95%)	*p* -Value
Anxiety a				
APACHE-II	1.274	1.033	0.976–1.094	0.259
Acute vs. chronic illness	6.408	3.849	1.356–10.926	0.011 b
Died in ICU vs. discharged	2.005	2.588	0.694–9.649	0.157
Nasogastric tube (+) vs. (−)	0.476	1.610	0.416–6.233	0.490
Depression c				
APACHE-II	3.158	1.074	0.993–1.163	0.076
Acute vs. chronic illness	7.980	6.148	1.744–21.675	0.005 b
Died in ICU vs. discharged	3.908	4.986	1.014–24.518	0.048 b
Nasogastric tube (+) vs. (−)	0.017	1.093	0.289–4.127	0.896

Abbreviations: APACHE, Acute Physiology and Chronic Health Evaluation; ICU, intensive care unit.

a -2log likelihood = 95.037.

b Statistically significant, *p* < 0.05.

c -2log likelihood = 75.705.

## Results of the Interviews

Two spouses (wife and husband) and two daughters accepted the interview. Three of the interviewees' patients died in the ICU during this study. The fourth patient was bedbound and discharged to the palliative clinic from the ICU. The two daughters were healthcare providers working in the same clinic where this study was held and familiar with the ICU setting. The interviewees are lettered from A to D ( Table 5 ). All interviewees stated having difficulty in understanding and remembering what was told. The common feelings of all the interviewees were desperation and fear. The quotations from the interviews are presented in the discussion section.

**Table 5:** Characteristics of the interviewees

Interviewee	Acute illness	Familiarity with ICU setting	Outcome
A	+	+	Death
B	−	+	Death
C	+	−	Discharged
D	−	−	Death

Abbreviation: ICU, intensive care unit.

## Discussion

The study's results indicated a considerably high prevalence of anxiety (52.4%) and depression (67.9%) symptoms among the family members of critically ill patients. The severity of the disease, having a nasogastric tube inserted into the patient, and acute developed illness were associated with symptoms of both anxiety and depression. Among these factors, acute illness and the anticipated death of the patient were independent factors for the symptoms of anxiety and depression.

Previous reports have shown a prevalence of anxiety symptoms varying from 35.9 to 78% and depression symptoms varying from 16 to 71.8% in family members of ICU patients. 1 11 17 18 19 These highly variable results from different centers might be affected by many issues, including regional and cultural differences, religious beliefs, environment-related factors…etc. 20 21 In addition to these regional differences, patient- or family-related factors may also affect mood. Earlier studies demonstrated that the young age of the patient and family member, low educational level and female gender of the family member, high severity scores, spouses, acute illness, and death of the patient were found to be associated with anxiety and depressive symptoms. 1 2 11 18 19 This study found that the family members of patients with high mortality rates and acute disease were more likely to present anxiety and depression symptoms, suggesting that a sudden and severe illness can produce massive emotional stress. Nevertheless, our results were unable to demonstrate any difference in terms of age, gender, intimacy, and educational level. The possible explanation for the age factor may be that the patients in this study were relatively older than those in the other studies. Furthermore, although the median age of family members with anxiety was lower than those without, this difference was not statistically significant. Additionally, the inconsistency in the intimacy factor might be due to the limited number of spouses participating in the study.

Consistent with the literature, this research found that acute illness is associated with anxiety and depressive symptoms. 2 For an acute event, panicking and not having time to say goodbye to a loved one in an unexpected situation may contribute to anxiety and depression. 22 Additionally, most family members experience a sensation of unreality and confusion when they first see their relative in the ICU. 23 In the first stage, the family member is not able to understand what is happening, as one of our interviewees described this condition:

“It was like falling from the top of a mountain”. (Interviewee A)

Moreover, the willingness to do anything for the loved one but not being able to may lead to anxiety and agitation, as in the expression of an interviewee:

“I do not know what to do. I am nervous because I cannot do anything.” (Interviewee C)

The results of the present study also accord with the literature that anxiety and depression symptoms were related to high disease severity scores and calculated mortality rates. This result can be interpreted as the anticipation of death might have raised anxiety and depression. 1 11 This situation was expressed by one of the interviewees as follows:

“I was hopeful for my father's recovery for a little while, but a part of me always knew this would not be possible. Finally, I accepted the situation and now trying to prepare myself for the inevitable ending with a feeling of deep sorrow.” (Interviewee B)

Furthermore, being well aware of the process and results of the clinical situation may cause a feeling of hopelessness. The healthcare provider whose father was admitted to the ICU during the course of this study stated that:

“The most intense feeling is desperation. I am helping everyone who needs me with all my knowledge and skill, but unfortunately, I cannot help my father.”. (Interviewee B)

One of the most significant issues in ICU care is the poor quality of communication between clinicians and family members. A previous study stated that half of the family members had inadequate communication with physicians. 24 A possible reason for this may be the negative effect of the anxiety and depression symptoms on the perception capacity. 2 Similarly to the previous findings, all of the interviewees in our study stated that they experienced difficulty in understanding and remembering what was told. Two interviewees expressed this phenomenon as follows:

“Sometimes I ask my colleagues to inform me as if they are explaining it to a 5-year-old child.” (Interviewee B)

“Everything you tell me about what you are doing to treat my life mate is confusing! I do not understand any of them, nor do I even want to know anything. I only need you to tell me, will he live or die?” (Interviewee D)

Many invasive procedures are performed in ICUs, and most patients have an endotracheal tube, feeding, or urinary catheters for treatment and follow-up. In a previous study, having an endotracheal tube was shown to be associated with complicated grief six months after death. 25 Similarly, an interesting finding of our study was that family members who saw their patients with nasogastric tubes showed more anxiety and depression symptoms than others. All the interviewees were concerned about “dangling tubes” on their relatives. However, there were two distinct explanations for their concern. While interviewees C and D (who were not familiar with the ICU environment) believed these tubes might cause pain, on the other hand, interviewees A and B (the two healthcare providers) mainly worried about the progression of the illness.

The study has several limitations. First, it was conducted in a single center; however, in the end, the findings have provided us with real benefits and positive ideas to improve institutional quality. Second, the study did not follow a particular phenomenological research method. However, we believe that direct quotations from the interviewees' statements can help empathize with family members. Another point that should be mentioned is that one of the authors of this article is one of the interviewees, which can be interpreted as a bias.

## Conclusion

The prevalence of anxiety and depression symptoms is significantly high in the family members of ICU patients. The severity of the disease, having a nasogastric tube, acute developed illness, and anticipated death are associated with symptoms of both anxiety and depression. The common feelings of family members are desperation and fear. Besides, family members can have difficulty in understanding and remembering what was told, along with anxiety and depressive symptoms. Awareness of this emotional stress can help develop interventions and attitudes to alleviate symptom burden. It can also contribute to the improvement of communication between physicians and family members.

## References

[1] French FAMIREA study group Pochard F Darmon M Fassier T Symptoms of anxiety and depression in family members of intensive care unit patients before discharge or death. A prospective multicenter study J Crit Care 2005 20 01 90 96 16015522 10.1016/j.jcrc.2004.11.004

[2] French FAMIREA Group Pochard F Azoulay E Chevret S Symptoms of anxiety and depression in family members of intensive care unit patients: ethical hypothesis regarding decision-making capacity Crit Care Med 2001 29 10 1893 1897 11588447 10.1097/00003246-200110000-00007

[3] Anderson W G Arnold R M Angus D C Bryce C L Passive decision-making preference is associated with anxiety and depression in relatives of patients in the intensive care unit J Crit Care 2009 24 02 249 254 19327283 10.1016/j.jcrc.2007.12.010

[4] FAMIREA Study Group Azoulay E Pochard F Kentish-Barnes N Risk of post-traumatic stress symptoms in family members of intensive care unit patients Am J Respir Crit Care Med 2005 171 09 987 994 15665319 10.1164/rccm.200409-1295OC

[5] Schmidt M Azoulay E Having a loved one in the ICU: the forgotten family Curr Opin Crit Care 2012 18 05 540 547 22914431 10.1097/MCC.0b013e328357f141

[6] Netzer G Sullivan D R Recognizing, naming, and measuring a family intensive care unit syndrome Ann Am Thorac Soc 2014 11 03 435 441 24673699 10.1513/AnnalsATS.201309-308OT PMC4028736

[7] Saeid Y Salaree M M Ebadi A Moradian S T Family intensive care unit syndrome (FICUS): an integrative review Iran J Nurs Midwifery Res 2020 25 05 361 368 33344205 10.4103/ijnmr.IJNMR_243_19 PMC7737832

[8] Davidson J E Jones C Bienvenu O J Family response to critical illness: postintensive care syndrome-family Crit Care Med 2012 40 02 618 624 22080636 10.1097/CCM.0b013e318236ebf9

[9] American College of Critical Care Medicine Task Force 2004-2005, Society of Critical Care Medicine Davidson J E Powers K Hedayat K M Clinical practice guidelines for support of the family in the patient-centered intensive care unit: American College of Critical Care Medicine Task Force 2004-2005 Crit Care Med 2007 35 02 605 622 17205007 10.1097/01.CCM.0000254067.14607.EB

[10] Kang J Cho Y J Choi S State anxiety, uncertainty in illness, and needs of family members of critically ill patients and their experiences with family-centered multidisciplinary rounds: a mixed model study PLoS One 2020 15 06 e0234296 32516349 10.1371/journal.pone.0234296 PMC7282650

[11] Köse I Zincircioğlu Ç Öztürk Y K Factors affecting anxiety and depression symptoms in relatives of intensive care unit patients J Intensive Care Med 2016 31 09 611 617 26168801 10.1177/0885066615595791

[12] Aydemir O Guvenir T Kuey L Kultur S Validity and reliability of Turkish version of the hospital anxiety and depression scale Turk Psikiyatr Derg 1997 8 280 287

[13] Zigmond A S Snaith R P The hospital anxiety and depression scale Acta Psychiatr Scand 1983 67 06 361 370 6880820 10.1111/j.1600-0447.1983.tb09716.x

[14] Kentish-Barnes N Lemiale V Chaize M Pochard F Azoulay E Assessing burden in families of critical care patients Crit Care Med 2009 37(10, Suppl):S448 S456 20046134 10.1097/CCM.0b013e3181b6e145

[15] Knaus W A Draper E A Wagner D P Zimmerman J E APACHE II: a severity of disease classification system Crit Care Med 1985 13 10 818 829 3928249

[16] Groenewald T A phenomenological research design illustrated Int J Qual Methods 2004 3 01 42 55

[17] McAdam J L Fontaine D K White D B Dracup K A Puntillo K A Psychological symptoms of family members of high-risk intensive care unit patients Am J Crit Care 2012 21 06 386 393, quiz 394 23117902 10.4037/ajcc2012582

[18] McAdam J L Dracup K A White D B Fontaine D K Puntillo K A Symptom experiences of family members of intensive care unit patients at high risk for dying Crit Care Med 2010 38 04 1078 1085 20124890 10.1097/CCM.0b013e3181cf6d94

[19] Anderson W G Arnold R M Angus D C Bryce C L Posttraumatic stress and complicated grief in family members of patients in the intensive care unit J Gen Intern Med 2008 23 11 1871 1876 18780129 10.1007/s11606-008-0770-2 PMC2585673

[20] Kleinman A Culture and depression N Engl J Med 2004 351 10 951 953 15342799 10.1056/NEJMp048078

[21] Kirmayer L J Cultural variations in the clinical presentation of depression and anxiety: implications for diagnosis and treatment J Clin Psychiatry 2001 62 13 22 28, discussion 29–30 11434415

[22] Cooper R A Segrin C When goodbyes matter: the conditional relationship between final conversations and symptoms of depression Death Stud 2023 47 05 585 591 35984769 10.1080/07481187.2022.2112319

[23] McKiernan M McCarthy G Family members' lived experience in the intensive care unit: a phenomenological study Intensive Crit Care Nurs 2010 26 05 254 261 20674362 10.1016/j.iccn.2010.06.004

[24] Azoulay E Chevret S Leleu G Half the families of intensive care unit patients experience inadequate communication with physicians Crit Care Med 2000 28 08 3044 3049 10966293 10.1097/00003246-200008000-00061

[25] Kentish-Barnes N Chaize M Seegers V Complicated grief after death of a relative in the intensive care unit Eur Respir J 2015 45 05 1341 1352 25614168 10.1183/09031936.00160014

